# 2-(3-Oxo-1,3-dihydro­isobenzofuran-1-yl)­phthalazin-1(2*H*)-one

**DOI:** 10.1107/S1600536809011970

**Published:** 2009-04-08

**Authors:** You-Lei Zhang, Yu-Jun Wu, Guo Peng, Hong Deng

**Affiliations:** a School of Chemistry and Environment, South China Normal University, Guangzhou 510006, People’s Republic of China

## Abstract

The reaction of 2-carboxy­benzaldehyde and hydrazine hydrate unexpectedly yielded the title compound, C_16_H_10_N_2_O_3_, which comprises one phthalide ring, one phthalazine system and a chiral centre. The phthalide unit is almost perpendicular to the phthalazine system, forming a dihedral angle of 87.1 (3)°. The packing is governed by weak C—H⋯O hydrogen-bonding inter­actions, forming layers parallel to the *ab* plane.

## Related literature

For general background to non-covalent inter­actions, see: Bernstein *et al.* (1995 ▶); Roesky & Andruh (2003 ▶). For related compounds, see: Nelson *et al.* (1982 ▶); Li *et al.* (2002 ▶); Özbey *et al.* (1998 ▶).
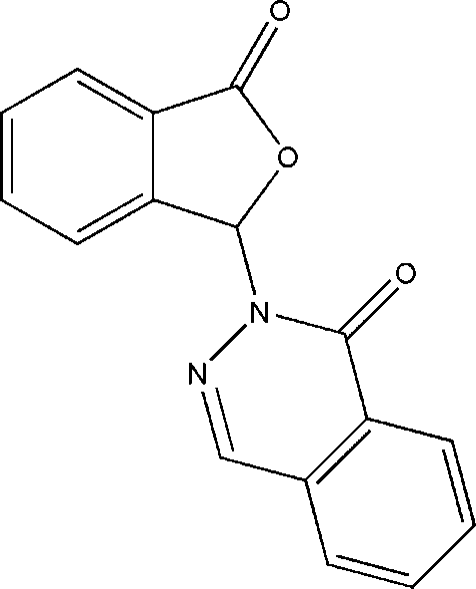


## Experimental

### 

#### Crystal data

C_16_H_10_N_2_O_3_
*M* *_r_* = 278.26
Triclinic, 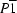

*a* = 7.2356 (2) Å
*b* = 8.0369 (2) Å
*c* = 11.1686 (4) Å
α = 80.047 (2)°
β = 86.093 (2)°
γ = 88.6550 (10)°
*V* = 638.17 (3) Å^3^
*Z* = 2
Mo *K*α radiation
μ = 0.10 mm^−1^
*T* = 296 K
0.20 × 0.18 × 0.15 mm

#### Data collection

Bruker APEXII area-detector diffractometer
Absorption correction: none
6746 measured reflections
2295 independent reflections
1626 reflections with *I* > 2σ(*I*)
*R*_int_ = 0.030

#### Refinement

*R*[*F* ^2^ > 2σ(*F* ^2^)] = 0.039
*wR*(*F* ^2^) = 0.110
*S* = 1.02
2295 reflections
190 parameters
H-atom parameters constrained
Δρ_max_ = 0.14 e Å^−3^
Δρ_min_ = −0.17 e Å^−3^

### 

Data collection: *APEX2* (Bruker, 2004 ▶); cell refinement: *SAINT* (Bruker, 2004 ▶); data reduction: *SAINT*; program(s) used to solve structure: *SHELXS97* (Sheldrick, 2008 ▶); program(s) used to refine structure: *SHELXL97* (Sheldrick, 2008 ▶); molecular graphics: *XP* in *SHELXTL* (Sheldrick, 2008 ▶); software used to prepare material for publication: *SHELXL97*.

## Supplementary Material

Crystal structure: contains datablocks I, global. DOI: 10.1107/S1600536809011970/rz2283sup1.cif

Structure factors: contains datablocks I. DOI: 10.1107/S1600536809011970/rz2283Isup2.hkl

Additional supplementary materials:  crystallographic information; 3D view; checkCIF report

## Figures and Tables

**Table 1:** Hydrogen-bond geometry (Å, °)

*D*—H⋯*A*	*D*—H	H⋯*A*	*D*⋯*A*	*D*—H⋯*A*
C16—H16⋯O3^i^	0.93	2.41	3.2935 (19)	158
C4—H4⋯O1^ii^	0.93	2.54	3.215 (2)	130

Symmetry codes: (i) 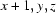; (ii) 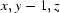.

**Fig. 1.**
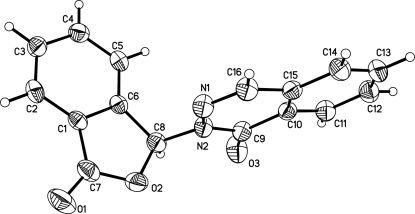
The molecular structure showing the atom-numbering scheme. Displacement ellipsoids drawn at the 30% probability level.

**Fig. 2.**
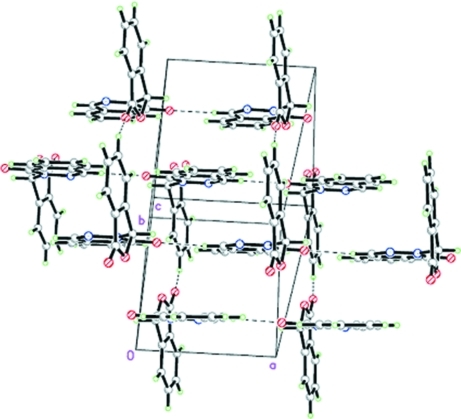
View of a supramolecular network of the title structure. Intemolecular hydrogen bonds are shown as dashed lines.

## supplementary crystallographic information

### Comment

Hydrogen bonding as an important type of non-covalent interaction plays a great role in supramolecular chemistry and material sciences (Bernstein *et al.*, 1995). Hydrogen bonding between solvent molecules and heterocycle compounds containing O or N donors has been confirmed to be a useful and powerful organizing force to form supramolecules (Roesky & Andruh, 2003). It has been reported (Nelson *et al.*, 1982) that the reaction of 2,6-diacetylpyridine and 1,2-phenylenediamine can form benzimidazole groups *via* oxidative dehydrogenation. Recently, in the reaction of 5-bromo-2-hydroxybenzaldehyde and 1,2-phenylenediamine in the presence of anhydrous ethanol solution a benzimidazole derivate have also been isolated (Li *et al.*, 2002). In this paper, we chose 2-carboxybenzaldehyde and hydrazinehydrate as reagents and unexpectedly isolated the heterocyclic title compound.

The molecular structure of the title compound is shown in Fig. 1. The phthalazine system is almost planar with the N1—C9—C10—C11 and C14—C15—C16—N2 torsion angles of -175.9 (2) and 178.0 (2)°, respectively. The phthalide ring system is also almost planar, the O2—C8—C6—C5 torsion angle being -176.7 (2)°. The C8—N1 bond length (1.449 (2) Å) indicates single bond character, and the corresponding bond angles demonstrate the *sp*^3^ character of  the C8 atom (chiral centre). The C16—N2 bond length (1.287 (2) Å) in the phthalazine ring has double bond character and is shorter than that found in the azomethine group of a related compound (Özbey *et al.*, 1998). The phthalide ring system is almost perpendicular to the phthalazine ring system, the dihedral angle they form being 87.1 (3)°. In the crystal structure, the molecules are linked *via* weak C—H···O hydrogen bonding interactions forming layers parallel to the *ab* plane (Table 1, Fig. 2).

### Experimental

2-Carboxybenzaldehyde (0.30 g, 2 mmol) and hydrazinehydrate (0.050 g, 1 mmol) were added to methanol (20 ml), and the mixture was refluxed for 3 h at 80°C. The resulting yellow precipitate was filtered and recrystallized from a MeOH/DMSO (5:1 v/v) solution. Colourless lamellar crystals were obtained on slow evaporation of the solvents at room temperature.

### Refinement

All H atoms were placed in calculated positions and were refined using a riding model, with (C—H = 0.93-0.96 Å, O—H = 0.82 Å), and with *U*_iso_(H) = 1.2 *U*_eq_(C) or  1.5 *U*_eq_(C, O) for methyl and hydroxy H atoms.

### Crystal data

**Table:**

C_16_H_10_N_2_O_3_	*Z* = 2
*M**_r_* = 278.26	*F*(000) = 288
Triclinic, *P*1	*D*_x_ = 1.448 Mg m^−^^3^
Hall symbol: -P 1	Mo *K*α radiation, λ = 0.71073 Å
*a* = 7.2356 (2) Å	Cell parameters from 2295 reflections
*b* = 8.0369 (2) Å	θ = 2.6–25.2°
*c* = 11.1686 (4) Å	µ = 0.10 mm^−^^1^
α = 80.047 (2)°	*T* = 296 K
β = 86.093 (2)°	Block, colourless
γ = 88.655 (1)°	0.20 × 0.18 × 0.15 mm
*V* = 638.17 (3) Å^3^	

### Data collection

**Table:**

Bruker APEXII area-detector diffractometer	1626 reflections with *I* > 2σ(*I*)
Radiation source: fine-focus sealed tube	*R*_int_ = 0.030
graphite	θ_max_ = 25.2°, θ_min_ = 2.6°
ω scans	*h* = −8→8
6746 measured reflections	*k* = −9→9
2295 independent reflections	*l* = −13→13

### Refinement

**Table:**

Refinement on *F*^2^	Primary atom site location: structure-invariant direct methods
Least-squares matrix: full	Secondary atom site location: difference Fourier map
*R*[*F*^2^ > 2σ(*F*^2^)] = 0.039	Hydrogen site location: inferred from neighbouring sites
*wR*(*F*^2^) = 0.110	H-atom parameters constrained
*S* = 1.02	*w* = 1/[σ^2^(*F*_o_^2^) + (0.0516*P*)^2^ + 0.0851*P*] where *P* = (*F*_o_^2^ + 2*F*_c_^2^)/3
2295 reflections	(Δ/σ)_max_ < 0.001
190 parameters	Δρ_max_ = 0.14 e Å^−^^3^
0 restraints	Δρ_min_ = −0.17 e Å^−^^3^

### Special details

**Table:**

Geometry. All e.s.d.'s (except the e.s.d. in the dihedral angle between two l.s. planes) are estimated using the full covariance matrix. The cell e.s.d.'s are taken into account individually in the estimation of e.s.d.'s in distances, angles and torsion angles; correlations between e.s.d.'s in cell parameters are only used when they are defined by crystal symmetry. An approximate (isotropic) treatment of cell e.s.d.'s is used for estimating e.s.d.'s involving l.s. planes.
Refinement. Refinement of *F*^2^ against ALL reflections. The weighted *R*-factor *wR* and goodness of fit *S* are based on *F*^2^, conventional *R*-factors *R* are based on *F*, with *F* set to zero for negative *F*^2^. The threshold expression of *F*^2^ > σ(*F*^2^) is used only for calculating *R*-factors(gt) *etc*. and is not relevant to the choice of reflections for refinement. *R*-factors based on *F*^2^ are statistically about twice as large as those based on *F*, and *R*- factors based on ALL data will be even larger.

### Fractional atomic coordinates and isotropic or equivalent isotropic displacement parameters (Å^2^)

**Table:**

	*x*	*y*	*z*	*U*_iso_*/*U*_eq_	
C1	0.2297 (2)	1.0612 (2)	0.05051 (17)	0.0452 (4)	
C2	0.2968 (2)	1.0522 (3)	−0.06731 (17)	0.0527 (5)	
H2	0.3332	1.1489	−0.1215	0.063*	
C3	0.3079 (2)	0.8959 (3)	−0.10149 (17)	0.0546 (5)	
H3	0.3514	0.8863	−0.1803	0.065*	
C4	0.2552 (3)	0.7527 (3)	−0.02004 (18)	0.0558 (5)	
H4	0.2650	0.6480	−0.0448	0.067*	
C5	0.1881 (2)	0.7618 (2)	0.09758 (17)	0.0504 (5)	
H5	0.1527	0.6652	0.1521	0.061*	
C6	0.1756 (2)	0.9188 (2)	0.13127 (15)	0.0433 (4)	
C7	0.2067 (3)	1.2059 (3)	0.1138 (2)	0.0609 (5)	
C8	0.1105 (2)	0.9693 (2)	0.24995 (17)	0.0522 (5)	
H8	−0.0224	0.9473	0.2655	0.063*	
C9	0.1102 (2)	0.7947 (2)	0.45282 (17)	0.0502 (5)	
C10	0.2253 (2)	0.7052 (2)	0.54673 (16)	0.0457 (4)	
C11	0.1443 (3)	0.6004 (2)	0.64863 (18)	0.0569 (5)	
H11	0.0167	0.5861	0.6574	0.068*	
C12	0.2554 (3)	0.5188 (3)	0.73568 (19)	0.0630 (5)	
H12	0.2024	0.4483	0.8036	0.076*	
C13	0.4459 (3)	0.5403 (3)	0.72353 (19)	0.0635 (6)	
H13	0.5191	0.4850	0.7838	0.076*	
C14	0.5268 (3)	0.6416 (3)	0.62413 (19)	0.0588 (5)	
H14	0.6546	0.6552	0.6167	0.071*	
C15	0.4170 (2)	0.7251 (2)	0.53311 (16)	0.0463 (4)	
C16	0.4925 (2)	0.8287 (3)	0.42442 (17)	0.0550 (5)	
H16	0.6202	0.8429	0.4162	0.066*	
N1	0.20803 (18)	0.8852 (2)	0.35304 (13)	0.0496 (4)	
N2	0.39749 (19)	0.9039 (2)	0.33708 (14)	0.0548 (4)	
O1	0.2366 (2)	1.35289 (19)	0.07887 (16)	0.0893 (5)	
O2	0.14034 (19)	1.14869 (17)	0.23133 (13)	0.0666 (4)	
O3	−0.05902 (16)	0.7908 (2)	0.45814 (13)	0.0764 (5)	

### Atomic displacement parameters (Å^2^)

**Table:**

	*U*^11^	*U*^22^	*U*^33^	*U*^12^	*U*^13^	*U*^23^
C1	0.0396 (9)	0.0427 (11)	0.0528 (11)	0.0040 (7)	−0.0141 (8)	−0.0033 (8)
C2	0.0451 (10)	0.0558 (13)	0.0519 (12)	−0.0009 (8)	−0.0109 (8)	0.0087 (9)
C3	0.0503 (11)	0.0639 (14)	0.0491 (11)	0.0075 (9)	−0.0068 (9)	−0.0081 (10)
C4	0.0593 (12)	0.0505 (12)	0.0596 (13)	0.0046 (9)	−0.0110 (9)	−0.0128 (10)
C5	0.0481 (10)	0.0456 (12)	0.0549 (12)	−0.0036 (8)	−0.0090 (8)	0.0020 (9)
C6	0.0337 (8)	0.0470 (11)	0.0483 (11)	0.0026 (7)	−0.0097 (7)	−0.0034 (8)
C7	0.0630 (13)	0.0509 (14)	0.0694 (15)	0.0109 (10)	−0.0240 (11)	−0.0056 (11)
C8	0.0421 (10)	0.0608 (13)	0.0534 (11)	0.0081 (8)	−0.0099 (8)	−0.0074 (9)
C9	0.0356 (9)	0.0667 (13)	0.0490 (11)	0.0013 (8)	−0.0035 (8)	−0.0116 (9)
C10	0.0389 (9)	0.0547 (11)	0.0452 (10)	0.0042 (8)	−0.0039 (8)	−0.0131 (9)
C11	0.0470 (10)	0.0650 (13)	0.0577 (12)	−0.0014 (9)	−0.0024 (9)	−0.0079 (10)
C12	0.0674 (13)	0.0620 (14)	0.0570 (13)	0.0014 (10)	−0.0053 (10)	−0.0029 (10)
C13	0.0685 (13)	0.0649 (14)	0.0577 (13)	0.0123 (10)	−0.0199 (10)	−0.0078 (11)
C14	0.0436 (10)	0.0724 (14)	0.0624 (13)	0.0080 (9)	−0.0150 (9)	−0.0134 (11)
C15	0.0385 (9)	0.0541 (11)	0.0490 (11)	0.0045 (8)	−0.0075 (8)	−0.0157 (9)
C16	0.0314 (9)	0.0761 (14)	0.0574 (12)	−0.0005 (8)	−0.0052 (8)	−0.0105 (10)
N1	0.0320 (7)	0.0707 (11)	0.0447 (9)	0.0051 (7)	−0.0048 (6)	−0.0061 (7)
N2	0.0331 (8)	0.0770 (12)	0.0527 (10)	0.0007 (7)	−0.0038 (7)	−0.0068 (8)
O1	0.1198 (14)	0.0428 (10)	0.1065 (13)	0.0052 (8)	−0.0350 (10)	−0.0064 (9)
O2	0.0757 (9)	0.0616 (10)	0.0661 (10)	0.0228 (7)	−0.0169 (7)	−0.0194 (7)
O3	0.0307 (7)	0.1177 (13)	0.0730 (10)	−0.0009 (7)	−0.0059 (6)	0.0066 (9)

### Geometric parameters (Å, °)

**Table:**

C1—C6	1.376 (2)	C9—O3	1.223 (2)
C1—C2	1.384 (3)	C9—N1	1.382 (2)
C1—C7	1.463 (3)	C9—C10	1.464 (2)
C2—C3	1.374 (3)	C10—C15	1.395 (2)
C2—H2	0.9300	C10—C11	1.396 (3)
C3—C4	1.381 (3)	C11—C12	1.372 (3)
C3—H3	0.9300	C11—H11	0.9300
C4—C5	1.382 (3)	C12—C13	1.388 (3)
C4—H4	0.9300	C12—H12	0.9300
C5—C6	1.377 (3)	C13—C14	1.364 (3)
C5—H5	0.9300	C13—H13	0.9300
C6—C8	1.495 (2)	C14—C15	1.401 (2)
C7—O1	1.198 (2)	C14—H14	0.9300
C7—O2	1.371 (3)	C15—C16	1.431 (3)
C8—O2	1.440 (2)	C16—N2	1.287 (2)
C8—N1	1.449 (2)	C16—H16	0.9300
C8—H8	0.9800	N1—N2	1.3786 (18)
			
C6—C1—C2	121.43 (18)	O3—C9—C10	124.45 (17)
C6—C1—C7	108.00 (17)	N1—C9—C10	114.62 (15)
C2—C1—C7	130.55 (18)	C15—C10—C11	120.32 (16)
C3—C2—C1	117.82 (18)	C15—C10—C9	119.31 (16)
C3—C2—H2	121.1	C11—C10—C9	120.37 (16)
C1—C2—H2	121.1	C12—C11—C10	119.18 (18)
C2—C3—C4	120.73 (19)	C12—C11—H11	120.4
C2—C3—H3	119.6	C10—C11—H11	120.4
C4—C3—H3	119.6	C11—C12—C13	120.7 (2)
C3—C4—C5	121.39 (19)	C11—C12—H12	119.6
C3—C4—H4	119.3	C13—C12—H12	119.6
C5—C4—H4	119.3	C14—C13—C12	120.66 (19)
C6—C5—C4	117.77 (17)	C14—C13—H13	119.7
C6—C5—H5	121.1	C12—C13—H13	119.7
C4—C5—H5	121.1	C13—C14—C15	119.88 (18)
C1—C6—C5	120.86 (17)	C13—C14—H14	120.1
C1—C6—C8	108.75 (16)	C15—C14—H14	120.1
C5—C6—C8	130.38 (17)	C10—C15—C14	119.22 (18)
O1—C7—O2	120.9 (2)	C10—C15—C16	117.71 (16)
O1—C7—C1	130.7 (2)	C14—C15—C16	123.06 (17)
O2—C7—C1	108.36 (18)	N2—C16—C15	125.13 (16)
O2—C8—N1	110.25 (14)	N2—C16—H16	117.4
O2—C8—C6	104.41 (14)	C15—C16—H16	117.4
N1—C8—C6	114.15 (14)	N2—N1—C9	126.76 (14)
O2—C8—H8	109.3	N2—N1—C8	113.34 (14)
N1—C8—H8	109.3	C9—N1—C8	119.88 (14)
C6—C8—H8	109.3	C16—N2—N1	116.35 (15)
O3—C9—N1	120.92 (17)	C7—O2—C8	110.40 (15)

### Hydrogen-bond geometry (Å, °)

**Table:**

*D*—H···*A*	*D*—H	H···*A*	*D*···*A*	*D*—H···*A*
C16—H16···O3^i^	0.93	2.41	3.2935 (19)	158
C4—H4···O1^ii^	0.93	2.54	3.215 (2)	130

Symmetry codes: (i) *x*+1, *y*, *z*; (ii) *x*, *y*−1, *z*.
